# Novel Kinetic Models of Xylan Dissolution and Degradation during Ethanol Based Auto-Catalyzed Organosolv Pretreatment of Bamboo

**DOI:** 10.3390/polym10101149

**Published:** 2018-10-15

**Authors:** Jing Liu, Zhenggang Gong, Guangxu Yang, Lihui Chen, Liulian Huang, Yonghui Zhou, Xiaolin Luo

**Affiliations:** 1 College of Materials Engineering, Fujian Agriculture and Forestry University, Fuzhou 350002, China; shamo107@163.com (J.L.); LaoGongAY00@163.com (Z.G.); yanggx94@163.com (G.Y.); lihuichen@263.net (L.C.); hll65212@163.com (L.H.); 2 Department of Civil and Environmental Engineering, Brunel University London, Kingston Lane, Uxbridge UB8 3PH, UK

**Keywords:** kinetic models, xylan, susceptible dissolution degree (d_X_), ethanol based auto-catalyzed organosolv (EACO) pretreatment, bamboo

## Abstract

Due to the invalidity of traditional models, pretreatment conditions dependent parameter of susceptible dissolution degree of xylan (d_X_) was introduced into the kinetic models. After the introduction of d_X_, the dissolution of xylan, and the formation of xylo-oligosaccharides and xylose during ethanol based auto-catalyzed organosolv (EACO) pretreatments of bamboo were well predicted by the pseudo first-order kinetic models (R^2^ > 97%). The parameter of d_X_ was verified to be a variable dependent of EACO pretreatment conditions (such as solvent content in pretreatment liquor and pretreatment temperature). Based on the established kinetic models of xylan dissolution, the dissolution of glucan and the formation of degradation products (furfural and acetic acid) could also be empirically modeled (R^2^ > 97%). In addition, the relationship between xylan and lignin removal can provide guidance for alleviating the depositions of lignin or pseudo-lignin. The parameter of d_X_ derived novel kinetic models can not only be used to reveal the multi-step reaction mechanisms of xylan, but also control the final removal of main components in bamboo during EACO pretreatments, indicating scientific and practical significance for governing the biorefinery of woody biomass.

## 1. Introduction

Clean and efficient fractionation of main components (i.e., cellulose, hemicellulose and lignin) in the biorefinery process is the prerequisite for realizing high-value utilization of woody biomass [1,2]. Among various pretreatment methods (such as hot water, dilute acid, dilute alkali and steam explosion, etc.), organosolv pretreatment has become one of the most effective methods because of its efficient fractionation of non-cellulosic components (hemicellulose and lignin) in woody biomass [3,4]. According to different types of solvent, organosolv pretreatment can be categorized into low-boiling alcohol, high-boiling alcohol, small molecular organic acid and other solvents (acetone, tetrahydrofuran, dimethyl sulfoxide and γ-valerolactone, etc.) based pretreatments [5]. Alternatively, organosolv pretreatment can be classified as acid catalytic and auto-catalytic pretreatments depending on whether a mineral acid (e.g., sulfuric acid or hydrochloric acid) is added as a catalyst [6]. The disadvantages associated with high-boiling alcohol, small molecular organic acid and other solvents based acid catalyzed organosolv pretreatment, such as uncompetitive solvent cost, equipment corrosion and lignin condensation, make them less promising for industrial applications than low-boiling alcohol based auto-catalytic organosolv pretreatment [5,7,8]. In reference to the latter, ethanol-based auto-catalytic organosolv (EACO) pretreatment is superior to other low-boiling alcohol (e.g., methanol) based pretreatment by virtue of being less toxic and bio-derived [8], and it has demonstrated significant industrialization potentials (such as Alcell process [6]).

In the process of EACO pretreatment, most of hemicellulose and lignin can be dissolved in the liquid phase in the forms of oligosaccharides, monosaccharides and degradations (e.g., furfural and aromatic compounds), while cellulose is mainly retained in the solid phase [3,5,6,9], which could be converted into fuel ethanol subsequently by enzymatic hydrolysis and fermentation [10]. In the liquid phase, xylo-oligosaccharides (Xy-Olig) can be used as health care products to improve human immunity [11], while xylose and lignin degradations can be used to produce value-added xylitol [12] and aromatic chemicals (e.g., tetrahydrofuran and furan dicarboxylic acid, etc.) [13]. The processing conditions (such as ethanol content and pretreatment temperature) have a crucial influence on the key performance indicators of EACO pretreatments, which include the accessibility of cellulose to enzymes, recoveries of xylan derived sugar (Xy-Olig and xylose) and lignin degradations [14,15]. Therefore, it is necessary to study the dissolution and degradation kinetics of the main non-cellulosic components (xylan and lignin) during EACO pretreatment, in order to better understand the multi-step reaction mechanisms and control the process.

**Scheme 1:**
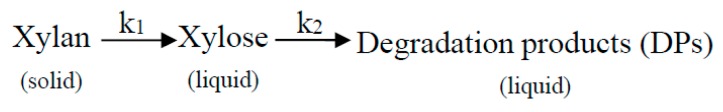
The reaction process of xylan involved in the Saeman model.

**Scheme 2:**
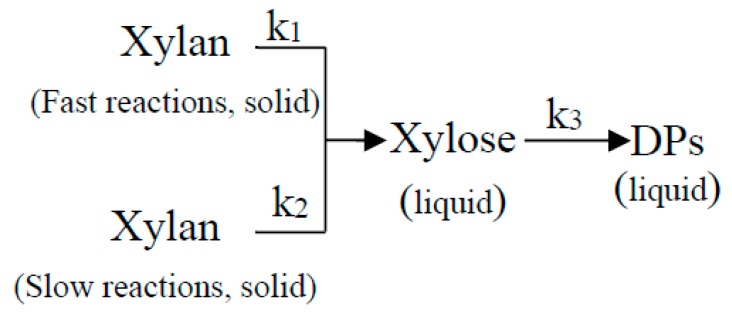
The reaction process of xylan involved in the two-phase model.

**Scheme 3:**
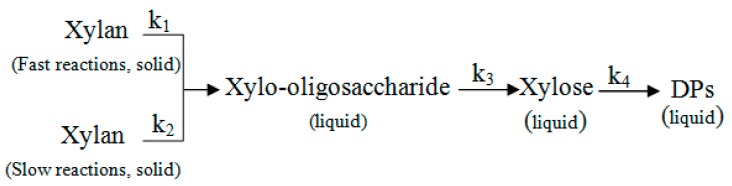
The reaction process of xylan involved in the modified two-phase model.

At present, there are numerous reports on the kinetics of lignin dissolution during EACO pretreatment, in which the transformation processes of lignin involve dissolution, condensation and deposition [5,6,16,17,18]. However, the dissolution and degradation kinetics of hemicellulose (mainly xylan) in this process has rarely been reported. Considering the reactions of xylan during acidic pretreatments (such as acid-catalyzed organosolv, dilute acid and hot water pretreatments) are similar to that of EACO pretreatment [5,6,8], the kinetics of xylan in these pretreatments are concluded in this work. Due to the invalidity of the simplest Saeman model (Scheme 1) [19], researchers had proposed a two-phase model (rapid and slow reactions, Scheme 2) to describe the dissolution behavior of xylan during acidic pretreatments [20,21]. Although this model had achieved some success in predicting xylan dissolution within the study conditions, the formation of Xy-Olig was neglected. Wei et al. [14] found that not only Xy-Olig were formed in the process of EACO pretreatment, their concentrations were also somewhat higher than that of xylose and other degradation products (e.g., furfural, etc.) under certain conditions. Aiming at overcoming the shortcomings of the above kinetic models, researchers further investigated the formation of Xy-Olig in a two-phase kinetic model (rapid and slow reactions, Scheme 3) during hot water [22] and ethanol-based acid-catalytic organosolv [23] pretreatments. These studies suggested that the ratio of xylan (“α”) undergone rapid hydrolysis is a variable independent of pretreatment conditions (such as solvent content in pretreatment liquor, pretreatment temperature and acid content, etc.), namely it is only related to the species of woody biomass. It is worth noting that the value of the “α” is also significantly affected by certain conditions (100~190 °C, 1~6% H_2_SO_4_ or HNO_3_, etc.) of hot water and dilute acid pretreatments [24,25,26]. Unfortunately, the relationship between the parameter of “α” and pretreatment conditions was not verified, though it plays a vital role in the thorough understanding of reaction mechanisms of xylan and process control during acid catalytic (dilute acid and acid-catalyzed organosolv) and auto-catalytic (hot water and EACO) pretreatments.

**Scheme 4:**
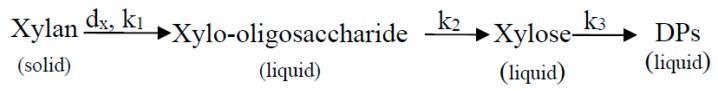
The parameter of susceptible dissolution degree of xylan (d_X_) introduced into the reaction process of xylan during EACO pretreatment.

This work aims to overcome the shortcomings of the aforementioned kinetic studies on the dissolution and degradation of xylan during EACO pretreatment through investigating the relationships between the ratio of fast-reacted xylan (“α”) and the pretreatment conditions (e.g., pretreatment temperature and ethanol content in pretreatment liquor). In the present study, the ratio of “rapidly hydrolyzed xylan” was initially defined as susceptible dissolution degree of xylan (d_X_). Based on the d_X_, the transformation process of xylan during EACO pretreatment of bamboo was rearranged as Scheme 4. The novel introduction of d_X_ into xylan dissolution and degradation kinetic models would help in achieving more accurate predictions, and hence serve as a viable tool for revealing the reaction mechanisms of xylan during EACO pretreatment and realizing better process control.

## 2. Materials and Methods

### 2.1. Materials

Bamboo chips were generously provided by Nanjing Forest Farm (Zhangzhou, China). After manual selection, the three-dimensional size of the bamboo chips is approximately 3.0 × 2.0 × 0.5 cm^3^. The glucan, xylan and Klason lignin contents of the bamboo chips were 40.7, 24.2 and 23.9% (w/w), respectively. Chemical standards (glucose, xylose, furfural and acetic acid, etc.) used for ion chromatography (IC) were purchased from Sigma-Aldrich Trading Co., Ltd. (Shanghai, China) and their purity are over 99% (w/w). Analytical grade ethanol used for pretreatments was obtained from Aladdin^®^ Reagent Co., Ltd. (Guangzhou, China).

### 2.2. Ethanol Based Auto-Catalyzed Organosolv (EACO) Pretreatment

EACO pretreatments were conducted in an oil bath digester (YYQ-10-1.25, Jiezhen Sci. & Tech., Nanjing, China). Pretreatment liquor and bamboo chips were placed in 1-L stainless-steel autoclaves, which can be fixed on the shelf of the big oil bath digester. The pretreatment temperature and time ranged from 443~473 *K* and 0~120 min, respectively. The content of ethanol in the pretreatment liquor was in the range of 0~98% (v/v). The ratio of pretreatment liquor (including ethanol and deionized water) to bamboo chips (on o.d. basis) for all pretreatments was fixed as 6:1 (mL:g). Upon completion of pretreatment in the digester, the autoclaves were immediately quenched by tap water for about 15 min. The resulted hydrolysate and solid fractions were separated by vacuum filtration on a Büchner funnel. The pretreatment hydrolysates were collected and stored in the refrigerator (4 °C) for further measurements. The pretreated solid substrates were washed one time with an equal amount of fresh pretreatment liquor and then washed three times with an equal amount of deionized water. The washed solid substrates were ground into smaller pieces using a laboratory mill (XY100, Yongkang Songqing Hardware Factory, Yongkang, China) and then vacuum dried at 35 °C for the measurement of solid substrate yield. 

### 2.3. Analysis of Pretreatment Hydrolysates and Solid Substrates

#### 2.3.1. Compositional Analysis of Raw Material and Substrates

The contents of main components in raw material (bamboo) and pretreated substrates were measured according to the method developed by National Renewable Energy Laboratory [27] with slight modifications. To promote the swelling of the sample by 72% (w/w) sulfuric acid, vacuum dried solid substrates were further ground in a mill (ZM 200, Retsch Inc., Haan, Germany) to pass a screen with a square opening size of 180 μm. 

After two steps of acid hydrolysis, the solid–liquid mixture was filtered on a Büchner funnel with filter paper. The filtrate was diluted and neutralized using deionized water and solid CaCO_3_, and then further filtered with 0.45 μm aqueous membrane. The final filtrate was analyzed by IC (Dionex ICS-5000 with Carbopac PA20 analytical column, Thermo Fisher Scientific-Dionex, Sunnyvale, CA, USA) for the measurements of monosaccharide concentrations (mainly glucose and xylose). The elution conditions of IC could be referred to the literature [28]. The solid residue was used to determine the content of Klason lignin. The glucan and xylan contents were mainly converted from the concentration of glucose and xylose in filtrate, respectively. 

The main component removal can be calculated by the following equation:

(1) $$R\;=\;\frac{C_{1}-Y\times C_{2}}{C_{1}}$$

where R is the component (glucan, xylan or Klason lignin) removal (%); C_1_ is the content of component in raw material (%, w/w); Y is the solid substrate yield (%, w/w, on o.d. basis of raw material); C_2_ is the content of component in solid substrate (%, w/w).

#### 2.3.2. Measurement of Degradations Concentration in Pretreatment Hydrolysates

The concentrations of Xy-Olig, xylose, furfural and acetic acid in pretreatment hydrolysates were also measured by IC (Dionex ICS-5000 with Carbopac PA20 analytical column, Thermo Fisher Scientific-Dionex, Sunnyvale, CA, USA) equipped with a dual system. 

For the determination of the concentration of degradation products (mainly furfural (F) and acetic acid (AA)), the pretreatment hydrolysates were previously diluted but without neutralization. For the measurement of xylose concentration, the pretreatment hydrolysate was pretreated with the methods shown in Section 2.3.1 and then analyzed by IC. For the total xylan derived sugar (oligo- and monosaccharides) concentration, the pretreatment hydrolysate was initially mixed with the previously configured sulfuric acid mother liquor to ensure the concentration of sulfuric acid in the pretreatment hydrolysate was 4% (w/w). The resulted pretreatment hydrolysate was then treated at 121 °C for 1 h. Prior to IC determination, the hydrolyzed pretreatment hydrolysates also need to be diluted, neutralized and filtered by following the above procedures (Section 2.3.1). The Xy-Olig concentration can be calculated based on the difference between the total xylan derived sugar and the xylose concentration.

The detailed elution conditions of IC with a dual system can also be referred to a previous report [28]. Specifically, the first system equipped with an amperometric detector, a guard column and an analytical column (Carbopac PA20, Thermo Fisher Scientific-Dionex, Sunnyvale, CA, USA) was used to measure Xy-Olig and xylose concentrations at 30 °C, while another system with a Supelcogel C-610H column and a UV detector was used to measure F and AA concentrations at 210 nm and 20 °C. 

## 3. Results and Discussion

### 3.1. Development of Kinetic Models

#### 3.1.1. Kinetic Model of Xylan Dissolution

The foregoing studies indicated that xylan dissolution followed the pseudo first-order kinetics during ethanol-based acid-catalytic organosolv pretreatment [18,23]. Based on the same hydrolysis mechanism (Brønsted acid catalyzed hydrolysis of glycosidic bonds) [5,8], the dissolution of xylan during EACO pretreatment can be simply described as:

(2) $$\frac{{\mathrm{dC}}_{\mathrm{Xy}-\mathrm{Poly}}}{\mathrm{dt}}=-k_{1}C_{\mathrm{Xy}-\mathrm{Poly}}$$

where C_Xy−Poly_ is the concentration (g/L) of residual xylan in the pseudo-homogenous system (assuming xylan exists in liquid phase) at pretreatment time of t (min); k_1_ presents the rate constant (min^−1^) of first reaction step shown in Scheme 4. 

$C_{\mathrm{Xy}-\mathrm{Poly}}^{0}$ refers to the initial concentration of xylan in the pseudo-homogenous system (g/L) without pretreatment. Based on the boundary condition (t = 0 min, C_Xy−Poly_ = $C_{\mathrm{Xy}-\mathrm{Poly}}^{0}$), integrating Equation (2) results in the time-dependent expression of C_Xy−Poly_ (Equation (3)).

(3) $$C_{\mathrm{Xy}-\mathrm{Poly}}=C_{\mathrm{Xy}-\mathrm{Poly}}^{0}\exp\left(-k_{1}t\right)$$

In the pseudo-homogenous system, the calculation of xylan removal (R_X_) shown in Equation (1) can be thus rearranged as:

(4) $$R_{X}\left(\%\right)=\left(\frac{C_{\mathrm{Xy}-\mathrm{Poly}}^{0}-C_{\mathrm{Xy}-\mathrm{Poly}}}{C_{\mathrm{Xy}-\mathrm{Poly}}^{0}}\right)\times 100=100\left(1-\frac{C_{\mathrm{Xy}-\mathrm{Poly}}}{C_{\mathrm{Xy}-\mathrm{Poly}}^{0}}\right)$$

By combining Equations (3) and (4), the relationship between R_X_ and k_1_ can be expressed as:

(5) $$\ln\left(1-\frac{R_{X}}{100}\right)=-k_{1}t$$

Under the specific pretreatment temperature and ethanol content in pretreatment liquor, ln(1 − R_X_/100) will linearly change with the pretreatment time (t) if Equation (5) is valid. However, under certain conditions (especially low ethanol content and high pretreatment temperature), there are almost no linear relationships between the above two parameters (Figure S1). The deviation indicated that either the above hypothesis for the development of kinetic model or the boundary condition for the integration of the Equation (2) is not in accordance with the fact. 

**Figure 1:**
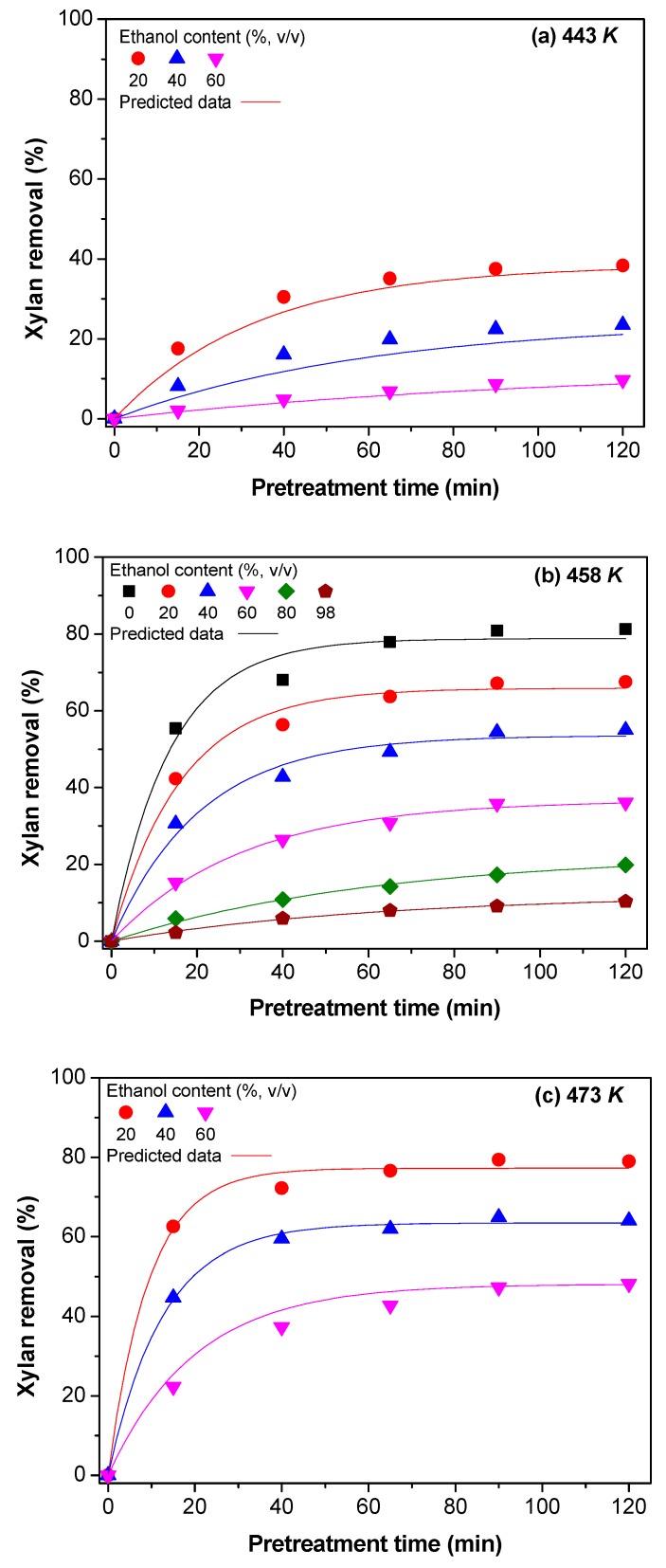
Measured and predicted xylan removal at EACO pretreatment temperature of (**a**) 443, (**b**) 458 and (**c**) 473 *K* with different ethanol content (%, v/v) in pretreatment liquor.

It can be seen from Figure 1 that, xylan is difficult to be completely dissolved from solid to liquid phase during EACO pretreatment with a specific ethanol content (%, v/v) and pretreatment temperature. For example, when the pretreatment time increased from 0 to 90 min, the R_X_ obtained at pretreatment temperature and ethanol content of 458 *K* and 40% (v/v) increased from 0% to 54.4% (Figure 1b). However, with a further extension of the pretreatment time to 120 min, the R_X_ was only slightly changed (55.0%). For EACO pretreatments with higher temperature and lower ethanol content, although the pretreatment time required to obtain the maximum R_X_ is reduced, corresponding R_X_ eventually tends to a level-off value (Figure 1c). Similar phenomena were also observed in the process of hot water [24] and dilute acid [25,26] pretreatments. Therefore, the above deviation (Figure S1) can be traced back to the boundary condition for the integration of Equation (2). In the pseudo-homogenous system, the initial (t = 0 min) concentration of xylan ($C_{\mathrm{Xy}-\mathrm{Poly}}^{0}$) should be the concentration of xylan corresponding to its level-off value obtained under specific conditions, but not the concentration of xylan in the feedstock. 

Based on these discussions, it is hypothesized that there is a pretreatment condition dependent parameter, i.e., susceptible dissolution degree of xylan (d_X_), that highly associates with the temperature and ethanol content of EACO pretreatment. When the pretreatment time is 0 min, the concentration of xylan existed in the pseudo-homogenous system will be $d_{X}C_{\mathrm{Xy}-\mathrm{Poly}}^{0}$. Therefore, Equations (2) and (4) can be modified as:

(6) $$\int_{d_{{\mathrm{xC}}_{\mathrm{Xy}-\mathrm{Poly}}^{0}}}^{C_{\mathrm{Xy}-\mathrm{Poly}}}\ln C_{\mathrm{Xy}-\mathrm{Poly}}{\mathrm{dC}}_{A}=-\int_{0}^{t}k_{1}\mathrm{dt}$$

(7) $$R_{X}\left(\%\right)=\frac{d_{x}C_{\mathrm{Xy}-\mathrm{Poly}}^{0}-C_{\mathrm{Xy}-\mathrm{Poly}}}{C_{\mathrm{Xy}-\mathrm{Poly}}^{0}}\times 100$$

The integral form of the Equation (6) is:

(8) $$C_{\mathrm{Xy}-\mathrm{Poly}}=d_{x}C_{\mathrm{Xy}-\mathrm{Poly}}^{0}\exp\left(-k_{1}t\right)$$

By taking Equation (8) into Equation (7), the time-dependent expression of R_X_ is obtained as follows:

(9) $$R_{X}\left(\%\right)=100d_{x}\left[1-\exp\left(-k_{1}t\right)\right]$$

#### 3.1.2. Kinetic Model of Xylo-Oligosaccharides Formation

Based on the assumption of the pseudo first-order kinetics [18,23], the formation of Xy−Olig during EACO pretreatment can be expressed as:

(10) $$\frac{{\mathrm{dC}}_{\mathrm{Xy}-\mathrm{Olig}}}{\mathrm{dt}}=1.136k_{1}C_{\mathrm{Xy}-\mathrm{Poly}}-k_{2}C_{\mathrm{Xy}-\mathrm{Olig}}$$

where C_Xy-Olig_ is the concentration of Xy-Olig in liquid phase (g/L); k_2_ presents the reaction rate constants of the second reaction step in Scheme 4 (min^−1^); 1.136 is the conversion coefficient of xylan to Xy-Olig (DP < 7). 

By introducing Equation (8) and the integral factor of exp(k_2_t) into Equation (10), this equation can be derived as:

(11) $$\exp\left(k_{2}t\right)\frac{{\mathrm{dC}}_{\mathrm{Xy}-\mathrm{Olig}}}{\mathrm{dt}}+k_{2}C_{\mathrm{Xy}-\mathrm{Olig}}\exp\left(k_{2}t\right)=1.136k_{1}d_{x}C_{\mathrm{Xy}-\mathrm{Poly}}^{0}\exp\left[\left(k_{2}-k_{1}\right)t\right]$$

By solving differential equation Equation (11) with its corresponding initial conditions (t = 0 min, $C_{\mathrm{Xy}-\mathrm{Olig}}^{0}$ = 0 g/L), the time-dependent expression of C_Xy-Olig_ is obtained as follows:

(12) $$C_{\mathrm{Xy}-\mathrm{Olig}}=\frac{1.136k_{1}d_{x}C_{\mathrm{Xy}-\mathrm{Poly}}^{0}}{k_{2}-k_{1}}\left[\exp\left(-k_{1}t\right)-\exp\left(-k_{2}t\right)\right]$$

#### 3.1.3. Kinetic Model of Xylose Formation

With the same assumption (pseudo first-order kinetics), the differential expression of xylose formation during EACO pretreatment can be expressed as:

(13) $$\frac{{\mathrm{dC}}_{\mathrm{Xy}-\mathrm{Mono}}}{\mathrm{dt}}=k_{2}C_{\mathrm{Xy}-\mathrm{Olig}}-k_{3}C_{\mathrm{Xy}-\mathrm{Mono}}$$

where C_Xy-Mono_ is the concentration of xylose in liquid phase (g/L); k_3_ presents the reaction rate constants of the third reaction step in Scheme 4 (min^−1^).

Based on Equation (12), integral factor exp(k_3_t) and boundary conditions (t = 0 min, C_Xy-Mono_ = 0 g/L), Equation (14) can be integrated as:

(14) $$C_{\mathrm{Xy}-\mathrm{Mono}}=\frac{1.136k_{1}k_{2}d_{x}C_{\mathrm{Xy}-\mathrm{Poly}}^{0}}{k_{2}-k_{1}}\left[\frac{\exp\left(-k_{1}t\right)-\exp\left(-k_{3}t\right)}{k_{3}-k_{1}}-\frac{\exp\left(-k_{2}t\right)-\exp\left(-k_{3}t\right)}{k_{3}-k_{2}}\right]$$

### 3.2. Determination of Kinetic Constants

#### 3.2.1. The d_x_ and Reaction Rate Constants

The d_X_ and reaction rate constants in kinetic models (Equations (9), (12) and (14)) could be fitted according to experimental data by a least square method in Microsoft Excel 2007 software to minimize the objective function ($\lim\sum_{i=1}^{n}{[f(x}_{i})-y_{i}]\to 0$). 

The d_X_ obtained at different EACO pretreatment conditions were listed in Table 1. It can be seen that on one hand, at a specific pretreatment temperature, the d_X_ gradually decreased with the increase of ethanol content (0~98%, v/v) in the pretreatment liquor. On the other hand, the d_X_ significantly increased with the increment of pretreatment temperature when ethanol content was fixed to a certain value. For example, with a pretreatment temperature of 458 *K*, the d_X_ increased from 0.12 to 0.79 when ethanol content decreased from 98 to 0% (v/v). With an ethanol content of 20% (v/v), pretreatment temperature (443~473 *K*) dependent increment of d_X_ (0.38 to 0.77) was also significant. This is possibly due to the fact that the removal of xylan during EACO pretreatment is mainly affected by the concentration of H^+^ in pretreatment liquor, which determines the hydrolysis rate of xylan to soluble sugars (oligosaccharides, monosaccharide) and degradations [14]. Under specific pretreatment temperature, the increase in ethanol content would inhibit the ionization of water to produce H^+^ [29]. However, with a certain ethanol content, high pretreatment temperature facilitates the ionization of water [5,8]. As a result, a high pretreatment temperature and a low ethanol content will be beneficial to the increase of the initial and/or real-time concentration of H^+^ in the pretreatment liquor. These speculations could be proved by the initial and real-time pH value of pretreatment liquor (Figure S2). Moreover, rapid ionization of water will also accelerate the hydrolysis rate of the acetyl group on the backbone of hemicellulose, which will produce acetic acid and thereby achieve an autocatalytic effect during EACO pretreatment [5,14,30]. Therefore, it can be confirmed again that during EACO pretreatment, the d_X_ is only dependent on the pretreatment temperature and the ethanol content in pretreatment liquor if the reaction time is long enough.

Similar to the d_X_, the pretreatment temperature and the ethanol content in the pretreatment liquor are also the key factors affecting the dissolution rate constant (k_1_) of xylan (Table 1). At a specific ethanol content (such as 20% (v/v)), the increase of pretreatment temperature significantly promoted the hydrolysis rate of xylan (0.11 min^−1^ at 473 *K* versus 0.03 min^−1^ at 443 *K*); however, with a certain pretreatment temperature, k_1_ gradually decreased with the increase of ethanol content. The effects of pretreatment temperature and ethanol content on the formation rates of xylo-oligosaccharides (k_2_) and xylose (k_3_) are similar to that of k_1_. 

**Figure 2:**
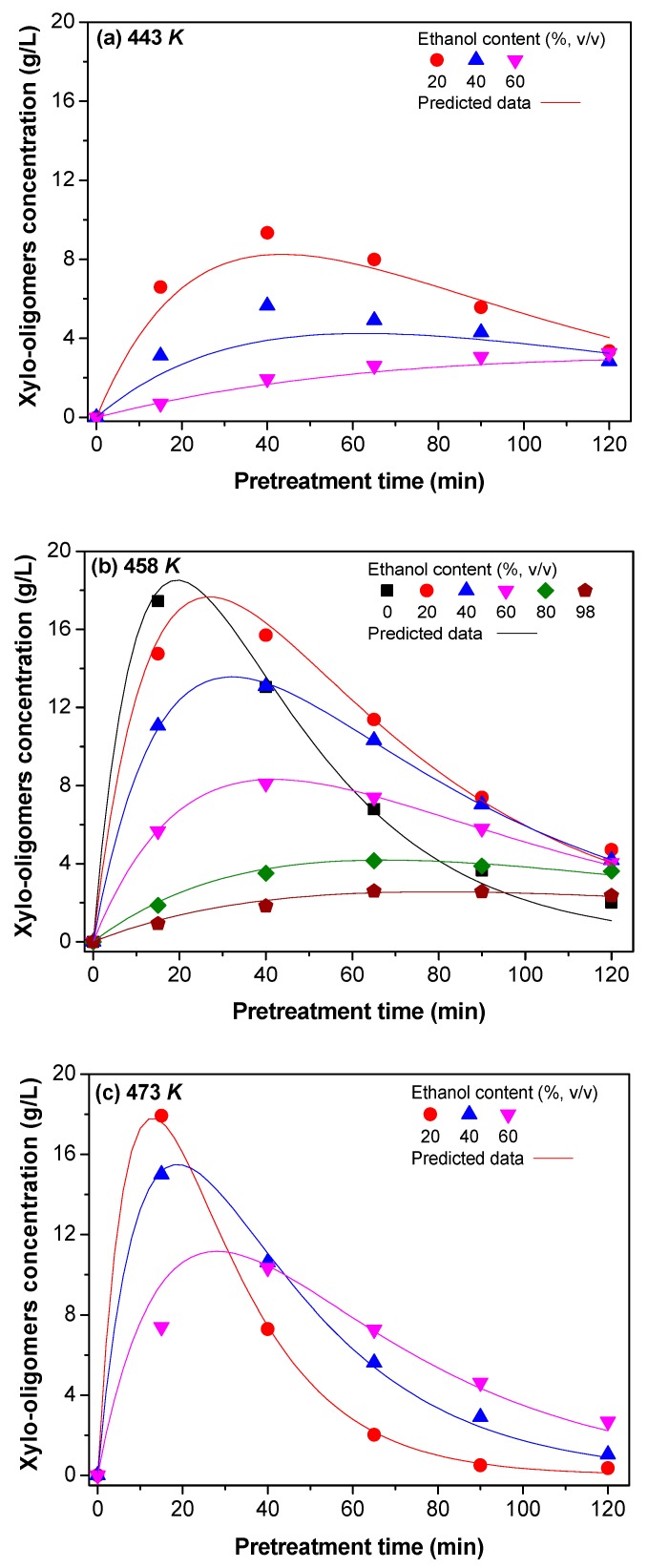
Measured and predicted xylo-oligosaccharides formation at EACO pretreatment temperature of (**a**) 443, (**b**) 458 and (**c**) 473 *K* with different ethanol content (%, v/v) in pretreatment liquor.

**Figure 3:**
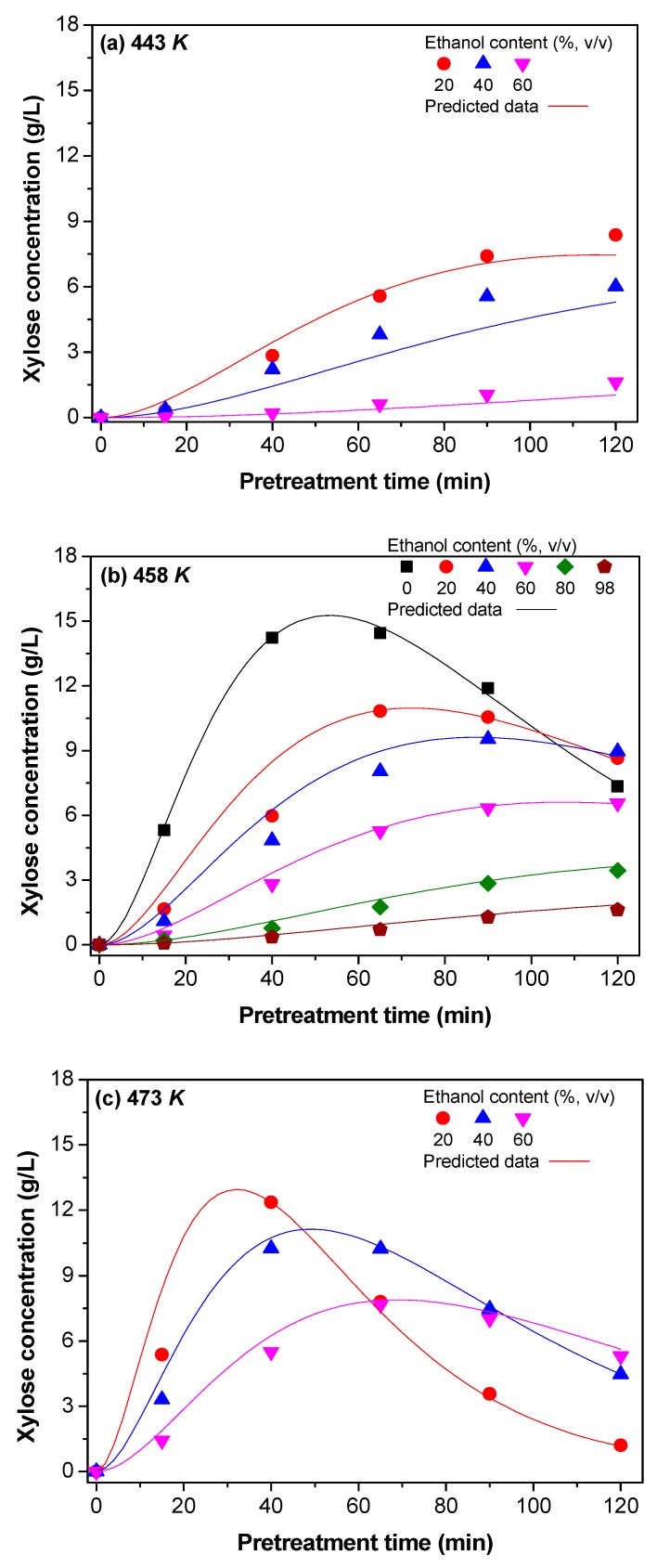
Measured and predicted xylose formation at EACO pretreatment temperature of (**a**) 443, (**b**) 458 and (**c**) 473 *K* with different ethanol content (%, v/v) in pretreatment liquor.

Based on the fitted d_X_ and reaction rate constants (k_1_, k_2_, and k_3_), R_X_, C_Xy-Olig_ and C_Xy-Mono_ presented in in Equations (9), (12) and (14) could be predicted. It was found that the measured data fell well on the prediction curves (Figure 1, Figure 2 and Figure 3). The determination coefficients (R^2^) of the regressions between experimental and predicted data were in the range of 0.975~0.995 (Figure S3), indicating the effectiveness of introducing d_X_ into kinetic models. 

#### 3.2.2. Activation Energy and Pre-Exponential Factor

According to the Arrhenius equation, the reaction rate constant k has the following relationship with the corresponding activation energy and pre-exponential factor:

(15) $$k=k_{0}\exp\left(-\mathrm{Ea}/\mathrm{RT}\right)$$

However, at a specific pretreatment temperature, the reaction rate constant k shown in Table 1 exponentially decreased with the increase of ethanol content during EACO pretreatment (fitting not shown). Therefore, Equation (15) can be modified as:

(16) $$k=k_{0}\frac{\exp\left(-\mathrm{Ea}/\mathrm{RT}\right)}{C_{\mathrm{Et}}^{\alpha }}$$

where k_0_, Ea and C_Et_ are the pre-exponential factor (min^−1^), activation energy (J/mol) and ethanol content in pretreatment liquor (%, v/v), respectively; α is the reaction order of ethanol content (%, v/v); R is the ideal gas constant (J/mol/*K*); and T is the pretreatment temperature (*K*). 

After logarithmic transformation, Equation (16) can be evolved as:

(17) $$\mathrm{lnk}={\mathrm{lnk}}_{0}-\frac{\mathrm{Ea}}{R}\frac{1}{T}-{\alpha \mathrm{lnC}}_{\mathrm{Et}}$$

The parameters in Equation (17) can be fitted by plotting lnk versus 1/T and lnC_Et_ with multivariate linear regression. The activation energy and other parameters for the dissolution of xylan and the formation of xylan-derived products (sugars and degradations) were shown in Table 2. The relatively low *p* values (<0.001, Table 2) indicate that this equation is significant and reliable to correlate the relationship between reaction rate constants and other parameters. The activation energy for the dissolution of xylan was 83.16 kJ/mol (Table 2), which is within the range (61~151 kJ/mol) of ethanol-based acid-catalytic organosolv pretreatment ([23] and references wherein). However, the activation energy for the degradation of xylose to furfural is considerably higher than that for the dissolution of xylan during EACO pretreatment. This phenomenon is similar to those for autohydrolysis (hot water) [24] and dilute acid [20,30] pretreatments of some non-wood biomass.

### 3.3. The Relationship between the d_X_ and Pretreatment Conditions

**Figure 4:**
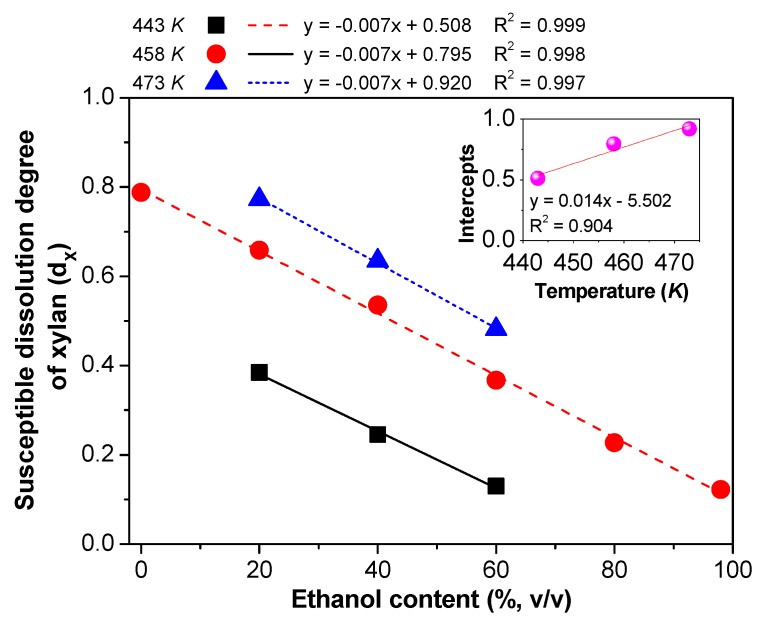
The correlations between d_x_ and ethanol content (%, v/v) in pretreatment liquor at different pretreatment temperatures.

The experimentally measured and model predicted dissolution of xylan in Figure 1 demonstrates that the value of d_X_ is only related to the pretreatment temperature and the ethanol content in the pretreatment liquor, but rather than the pretreatment time. Therefore, the d_X_ was separately plotted against the ethanol content at different pretreatment temperatures. It was found that these two parameters were in good linear relationships (R^2^ > 0.99). Therefore, for a particular pretreatment temperature, the relationship between d_X_ and the ethanol content can be expressed as:

(18) $$d_{X}={\delta C}_{\mathrm{Et}}+\gamma$$

where δ and γ refer to the slope and intercept of the fitted lines in Figure 4, respectively.

Interestingly, the slopes of the three fitting lines are basically the same, proving that the effects of ethanol content on d_X_ are consistent at different pretreatment temperatures. Therefore, it can be reasonably speculated that the intercepts of the fitting lines should be the pretreatment temperature induced potential removal of xylan during EACO pretreatment. The relationship between the intercepts of the fitting lines (big figure in Figure 4) and pretreatment temperature can be therefore fixed as:

(19) $$\gamma ={\rho T}_{A}+\lambda$$

where ρ and λ refer to the slope and intercept of another fitted line (small figure in Figure 4), respectively; T_A_ is the pretreatment temperature (K). 

Combining Equations (18) and (19) gives a quantitative mathematical relationship among d_X_, pretreatment temperature and ethanol content. It can be expressed as:

(20) $$d_{X}={\varphi C}_{\mathrm{Et}}+{\rho T}_{A}+\lambda$$

According to the fitting results in Figure 4, the value of δ, ρ and λ are −0.007, 0.014 and −5.502, respectively. A good correlation (R^2^ = 0.991, Figure S4) between the fitted d_X_ and the corresponding experimental value suggests the validity of this empirical model (Equation (20)).

### 3.4. Predicting the Formations of Furfural and Acetic Acid

Furfural (F) is an important degradation product (DP) of xylose [5,23,24,26], while the release of acetic acid (AA) from the acetyl groups in side chains of hemicellulose (mainly xylan) [31] lays the foundation for realizing the autocatalysis of EACO pretreatment. Therefore, accurate prediction of both DPs will be beneficial to controlling the overall process of EACO pretreatment. 

Similar to the H-factor [32] used in the commercial alkaline pulping industry, P-factor [31,33] has commonly used to unify the effects of temperature and time on hemicellulose dissolution during autohydrolysis (such as hot water) pretreatment. Since the average activation energy (126 kJ/mol) of xylan dissolution during hot water pretreatment [34] is much higher than this study, the P-factor used in this study [31,35] is simply modified with the corresponding activation energy (83.16 kJ/mol) obtained in the process of EACO pretreatment. It can be expressed as:

(21) $$\mathrm{Modified}\;P-\mathrm{factor}\;\left(\mathrm{MP}\right)=t\times \exp\left(\mathrm{Ea}/\left({\mathrm{RT}}_{0}\right)-\mathrm{Ea}/\left(R\left(T+T_{0}\right)\right)\right)$$

where T_0_ and T are reference temperature (373 *K*) and pretreatment temperature (*K*), respectively; t is the pretreatment time, min; Ea is the activation energy (83,157 J/mol) of xylan dissolution during EACO pretreatment; R is the ideal gas constant (J/mol/*K*). 

**Figure 5:**
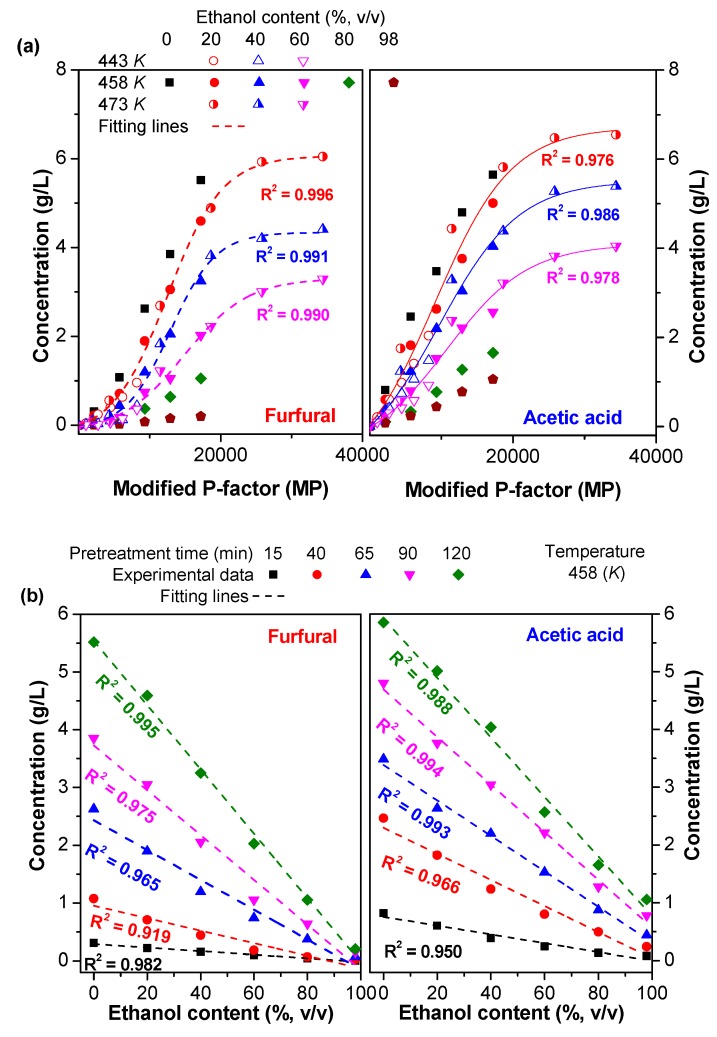
(**a**) Modified P-factor (MP) and (**b**) ethanol concentration dependent furfural and acetic acid concentration during EACO pretreatments.

At the specific ethanol content, the concentrations of DP (F or AA) were initially plotted against the corresponding MPs. It was found that the experimental data generated by different temperatures basically fell onto one curve and a Boltzmann function relationship was obtained (Figure 5a). Therefore, the relationship between the concentrations of DP (F or AA) and the corresponding MPs at a specific ethanol content can be expressed as:

(22) $$C_{\mathrm{DP}}\;\left(F_{1}{\;\mathrm{or}\;\mathrm{AA}}_{1}\right)\;=\lambda_{2}+\frac{\lambda_{1}-\lambda_{2}}{1+\exp\left[\left(\mathrm{MP}-{\mathrm{MP}}_{0}\right)/\Delta \mathrm{MP}\right]}$$

where λ_1_ and λ_2_ are the initial and final concentration (g/L) of DP shown in Boltzmann function; MP_0_ presents the value of MP at which DP (F and AA) concentration changed sharply; ∆MP is the change rate of MP with F or AA concentration. 

When t = 0 min, initial concentration of F or AA equals to 0 g/L. Thus, Equation (22) can be simplified as:

(23) $$C_{\mathrm{DP}}\;\left(F_{1}{\;\mathrm{or}\;\mathrm{AA}}_{1}\right)\;=\lambda_{2}\left[1-\frac{1}{1+\exp\left(\frac{\mathrm{MP}-{\mathrm{MP}}_{0}}{\Delta \mathrm{MP}}\right)}\right]$$

In addition, at a specific pretreatment temperature and time (so-called MP), the concentration of DP (F or AA) was linearly changed with the ethanol content (Figure 5b). Their relationship can be thus described as:

(24) $$C_{\mathrm{DP}}\;\left(F_{1}{\;\mathrm{or}\;\mathrm{AA}}_{1}\right)\;=-{\mathrm{BC}}_{\mathrm{Et}}+C$$

where B and C are constants. 

Considering the combined effects of MP (Equation (23)) and ethanol content (Equation (24)), the empirical prediction model for the concentration of DP (F or AA) during EACO pretreatment can be simply expressed as:

(25) $$C_{\mathrm{DP}}\;\left(F\;\mathrm{or}\;\mathrm{AA}\right)\;=\left[1-\frac{1}{1+\exp\left(\frac{\mathrm{MP}-{\mathrm{MP}}_{0}}{\Delta \mathrm{MP}}\right)}\right]\left(-{\delta C}_{\mathrm{Et}}+\varepsilon \right)$$

where δ and ε are constants.

The parameters in Equation (25) were fitted by the least square method and listed in Table 3. The predicted data by this empirical model was in a good agreement with experimental results (R^2^ > 0.97, Figure S5). Moreover, it can also be found that the MP_0_ corresponding to the rapid increase of the AA concentration is lower than that of F (Table 3). According to the discussion in Section 3.2, this may be mainly caused by two reasons: (1) the activation energy of xylan hydrolysis is lower than that of xylose dehydration to form F (Table 2); (2) the reaction rate constant (k_1_) of the first step reaction in Scheme 4 is much higher than that of the third step reaction (k_3_). These imply that the prediction results of this empirical model are consistent with the above kinetic models.

### 3.5. The Relationships between Xylan and Other Main Component Removal

#### 3.5.1. The Relationship between Xylan and Glucan Removal

**Figure 6:**
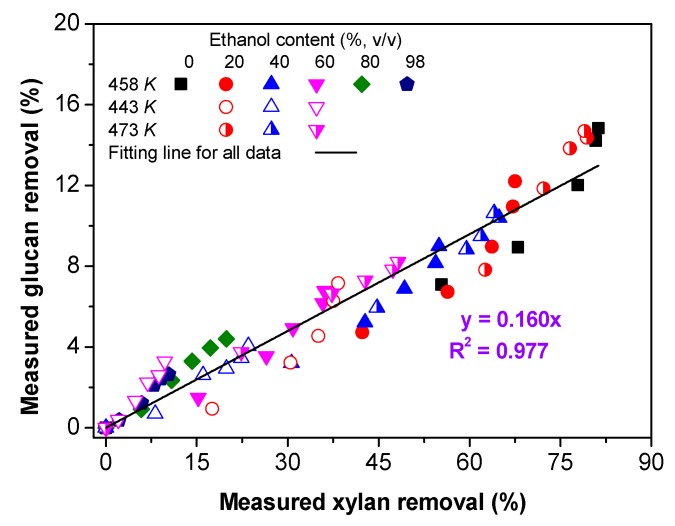
The relationship between measured xylan and glucan removal during EACO pretreatments.

Previous research under the same conditions [14] suggested that cellulose (mainly glucan) removal was significantly lower than that of hemicellulose (mainly xylan) during EACO pretreatment, but their relationship was barely correlated. Within the range of EACO pretreatment conditions carried out in this study, the glucan removal (R_G_) was linearly changed with the corresponding R_X_ (Figure 6). Since the slope of the fitting line is 0.160 when the intercept is fixed as 0, the prediction model of the R_G_ can be developed based on Equations (9), (16) and (20) with the corresponding parameters shown in Table 2 and Figure 4. It can be directly presented as:

(26) $$R_{G}\left(\%\right)=16\left(-0.007C_{\mathrm{Et}}+0.014T_{A}-5.502\right)\{1-\exp\left[-\left(2.72\times 10^{9}\times \frac{\exp\left(-83160/\mathrm{RT}\right)}{C_{\mathrm{Et}}^{0.88}}\right)t\right]\}$$

Due to the achievement of good prediction (R^2^ > 0.99, Figure S3a) of R_X_ by Equation (9) after the introduction of d_X_ and reliable linear correlation (R^2^ > 0.97, Figure 6) between R_G_ and R_X_, it can be reasonably assumed that Equation (26) is able to accurately predict the R_G_ during EACO pretreatments.

#### 3.5.2. The Relationship between Xylan and Klason Lignin Removal

The relationship between Klason lignin removal (R_L_) and R_X_ has been presented in Figure S6. It can be found that under the specific ethanol content (%, v/v), different pretreatment temperature induced R_L_ basically fell onto one curve, which apparently increased and then decreased with the increase of R_X_ during EACO pretreatments. The maximum R_L_ corresponds to an ethanol content of 60% (Figure S6), which is similar to the maximum solubility of ALCELL^®^ lignin in an acid-free ethanol-water system (60~70% (v/v)) [36]. In addition to the effect of H^+^ concentration on the degree of lignin degradation (such as homolytic cleavage of β-O-4 linkages, etc.) [5,15], this may be primarily related to the solubility parameters of the solvent system. Although the Hildebrand solubility parameter (δ_H_) of ethanol (26.0 (J/cm^3^)^−1/2^) is close to that of lignin (22.5 (J/cm^3^)^−1/2^), the δ_H_ of water (47.3 (J/cm^3^)^−1/2^) is far from lignin’s δ_H_ [5,29]. Therefore, an excessive or insufficient ethanol content in the solvent system will reduce the solubility of the degraded lignin in the pretreatment liquor. In the process of autocatalytic hydrothermal pretreatments (such as hot water and EACO pretreatments) with high temperature, degraded lignin fragments and carbohydrate degradation products (such as F) could undergo condensation and/or polymerization reactions to form condensed lignin [5,15,37] or pseudo-lignin [38], if the pretreatment liquor has a low solubility of lignin. Compared with the lignin fragments with smaller molecular weight, previous studies [29,39,40] also indicated that the condensed lignin or pseudo-lignin was more preferential to deposit on the surface of the solid substrate. These deposits could result in significant non-effective adsorption of enzymes and therefore inhibit the enzymatic conversion of cellulose in pretreated solid substrates [39,40]. 

Therefore, to improve the recovery of lignin and reduce the negative effect of these deposits on the enzymatic conversion of cellulose, it is necessary to balance the relationship between R_L_ and R_X_ during EACO pretreatment. Although the R_L_ and R_X_ at different ethanol content did not form a uniform function, the kinetic model of R_X_ can also be regarded as an effective tool to control the process of EACO pretreatment.

## 4. Conclusions

A novel parameter of susceptible dissolution degree of xylan (d_X_), which related to pretreatment temperature and ethanol content in pretreatment liquor, was introduced into the kinetic models of xylan dissolution and degradation during EACO pretreatments of bamboo. The introduction of d_X_ improved the validity of these kinetic models. The changes of other main components (glucan and Klason lignin) and degradation products (furfural and acetic acid) could also be well predicted or controlled based on these established kinetic models. For enhancing the role of EACO pretreatment in the biorefinery, these kinetic models will provide a fundamental basis for understanding the reaction mechanisms and realizing the process control and optimization.

## Figures and Tables

**Table 1:** The parameters of kinetic models shown in Equations (9), (12) and (14) for xylan dissolutions and degradation during EACO pretreatments.

Ethanol content (%, v/v)	Pretreatment temperature (*K*)											
	443				458				473			
	d_x_	k_1_	k_2_	k_3_	d_x_	k_1_	k_2_	k_3_	d_x_	k_1_	k_2_	k_3_
**0**	NA	NA	NA	NA	0.7878	0.0735	0.0344	0.0214	NA	NA	NA	NA
**20**	0.3844	0.0297	0.0175	0.0101	0.6586	0.0623	0.0199	0.0189	0.7723	0.1077	0.0530	0.0431
**40**	0.2450	0.0165	0.0156	0.0037	0.5355	0.0486	0.0185	0.0148	0.6346	0.0794	0.0336	0.0267
**60**	0.1293	0.0093	0.0046	0.0010	0.3670	0.0327	0.0169	0.0121	0.4812	0.0387	0.0243	0.0221
**80**	NA	NA	NA	NA	0.2272	0.0162	0.0135	0.0103	NA	NA	NA	NA
**98**	NA	NA	NA	NA	0.1218	0.0158	0.0098	0.0090	NA	NA	NA	NA

Note: the units of reaction rate constants (k_1_, k_2_ and k_3_) and d_x_ are min^−1^ and %, respectively; NA presents “NOT AVAILABLE”.

**Table 2:** The reaction activity energies and other parameters in kinetic models of xylan dissolutions and degradation during EACO pretreatments.

k	k_0_ (min^−1^)	Ea (kJ/mol)	α	F value	*p* value
**k_1_**	2.72 × 10^9^	83.16	0.88	59.1280	0.0000
**k_2_**	1.12 × 10^7^	68.19	0.63	20.1566	0.0010
**k_3_**	4.38 × 10^13^	126.84	0.69	18.2701	0.0006

**Table 3:** The parameters in the empirical model (Equation (25)) for predicting the formation of degradations (F or AA) during EACO pretreatments.

Degradation products (DPs)	Parameters in empirical model (Equation (40))			
	MP_0_ (min)	∆MP	δ	ε
**Furfural (F)**	12,984.6	3474.0	0.0760	7.4865
**Acetic acid (AA)**	10,933.0	4025.4	0.0689	7.8696

## Supplementary Materials

The following are available online at http://www.mdpi.com/2073-4360/10/10/1149/s1, Figure S1: The relationship between ln(1 − R_x_/100) and t at EACO pretreatment temperature of (a) 443, (b) 458 and (c) 473 *K*, Figure S2: pH value of pretreatment liquor at EW pretreatment temperature of (a) 443, (b) 458 and (c) 473 *K* with different ethanol content in pretreatment medium, Figure S3: Comparisons of measured and model predicted data under different conditions of EACO pretreatments: (a) xylan removal; (b) xylo-oligosaccharides and (c) xylose concentration, Figure S4: The relationship between experimentally measured “d_x_” and its predicted value, Figure S5: The relationships between measured and predicted concentration of degradation products: (a) F and (b) AA, Figure S6: The relationship between experimentally measured xylan and lignin removal.
