# Supplementary material for: Novel Kinetic Models of Xylan Dissolution and Degradation during Ethanol Based Auto-Catalyzed Organosolv Pretreatment of Bamboo
Source: Polymers (Basel). 2018 Oct 15;10(10):1149. doi: 10.3390/polym10101149 (PMC6403872; doi:10.3390/polym10101149)
Supplement: Supplementary file 1 [file polymers-10-01149-s001.pdf]

Supplementary Materials: Novel Kinetic Models of Xylan Dissolution and Degradation during Ethanol Based Auto-Catalyzed Organosolv Pretreatment of Bamboo

Jing Liu [1], **Zhenggang Gong** [1], **Guangxu Yang** [1], **Lihui Chen** [1], **Liulian Huang** [1], **Yonghui Zhou** [2,*] and **Xiaolin Luo** [1,*]

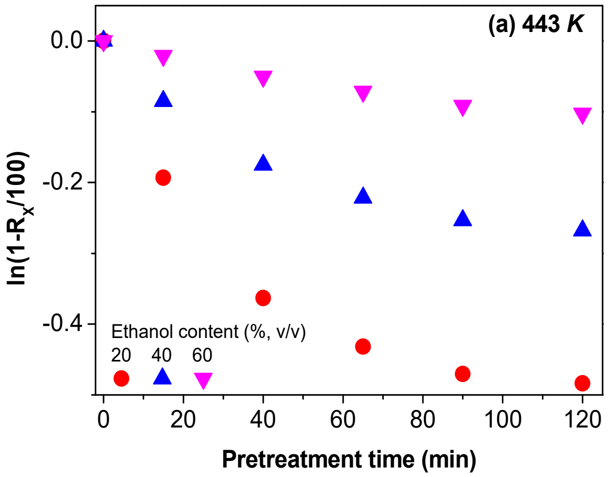

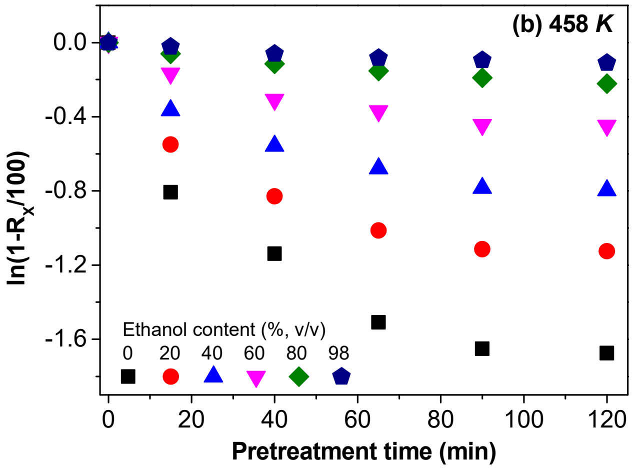

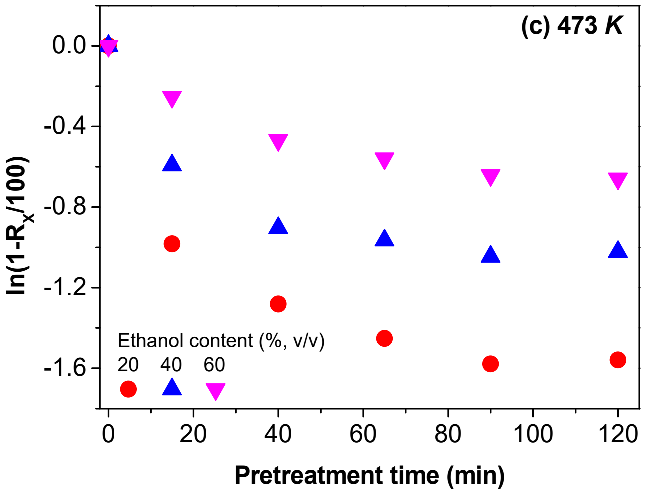

Figure S1 The relationship between ln(1-R$_x$/100) and t at EACO pretreatment temperature of (**a**) 443, (**b**) 458 and (**c**) 473 *K*.

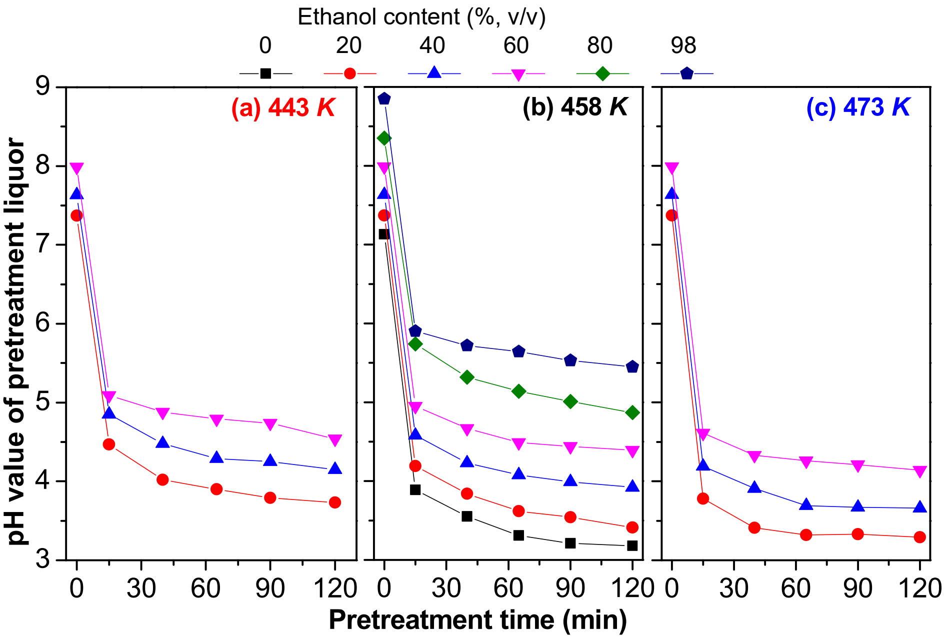

Figure S2 pH value of pretreatment liquor at EW pretreatment temperature of (**a**) 443, (**b**) 458 and (**c**) 473 *K* with different ethanol content in pretreatment medium.

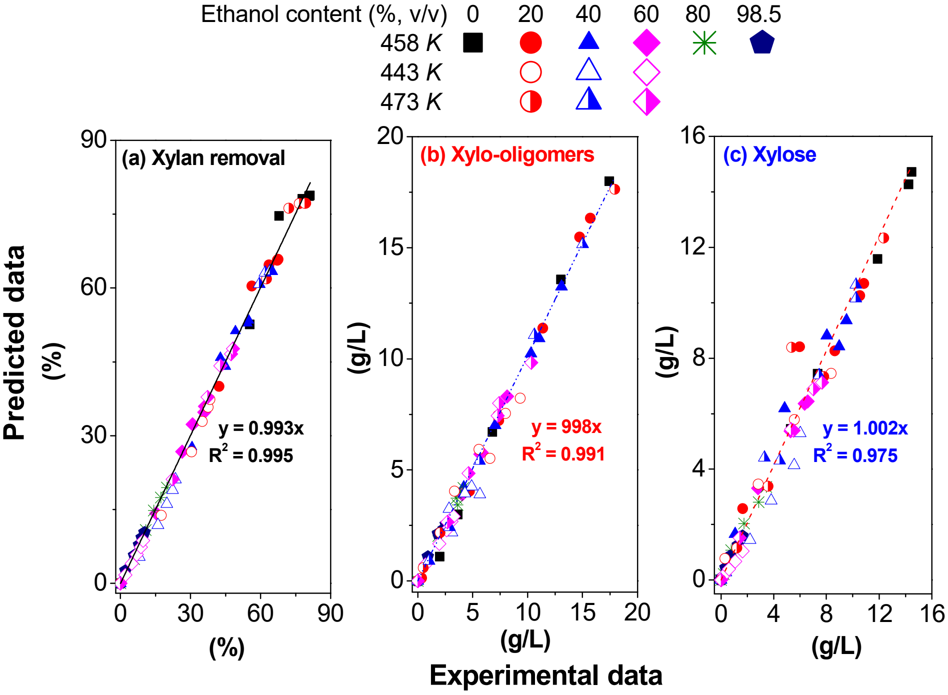

Figure S3 Comparisons of measured and model predicted data under different conditions of EACO pretreatments: (**a**) xylan removal; (**b**) xylo-oligosaccharides and (**c**) xylose concentration.

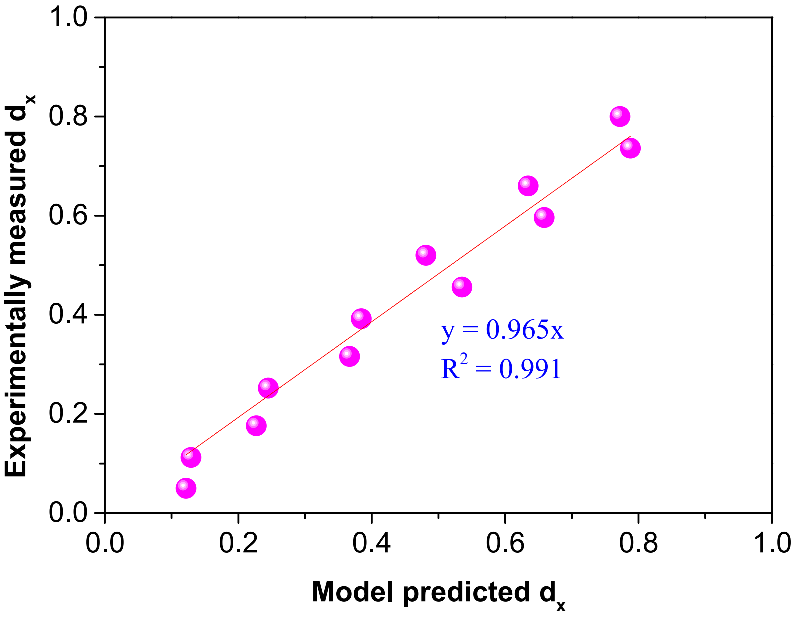

Figure S4 The relationship between experimentally measured "d_x" and its predicted value.

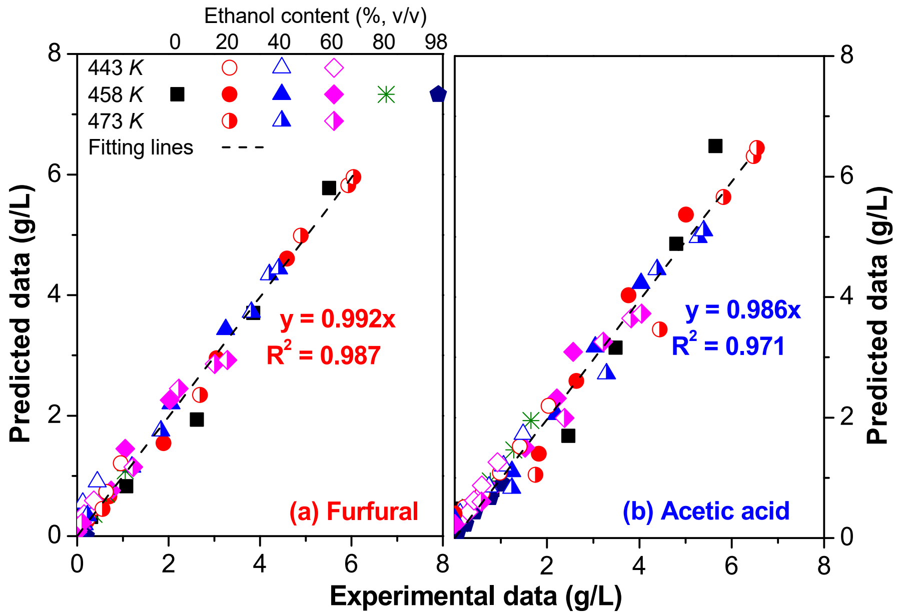

Figure S5 The relationships between measured and predicted concentration of degradation products: (**a**) F and (**b**) AA.

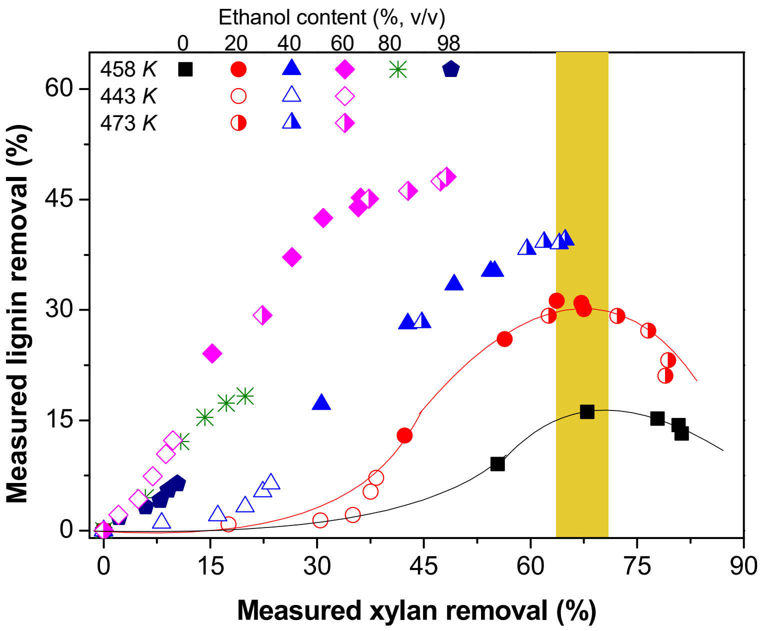

Figure S6 The relationship between experimentally measured xylan and lignin removal.
